# Correction: Predictors of food variety and food consumption scores of adolescents living in a rural district in Ghana

**DOI:** 10.1371/journal.pone.0307682

**Published:** 2024-07-18

**Authors:** Michael Akenteng Wiafe, Jessica Ayensu, Georgina Benewaa Yeboah

The Tables 1, 2 and 3 are formatted incorrectly. Please see the correct Tables 1, 2 and 3 here.

**Table 1:** Sociodemographic, food practices, food variety score and food consumption score of adolescents.

Variable	N (%)	Food variety score		Food consumption score			
		Mean ± Sd	p-value	Food insecure	Food secure	X^2^	p-value
**Sex**							
Male	70(51.1)	24.8±6.2	0.050	27(40.3)	43(61.4)	6.1	0.017
Female	67(48.9)	26.9±6.3		40(59.7)	27(38.6)		
**Community**							
Peri-urban	69(50.4)	26.1±7.3	0.573	26(38.8)	43(61.4)	7.0	0.010
Rural	68(49.6)	25.5±5.2		41(61.2)	27(38.6)		
**Educational status**							
**Participants**							
JHS	41(29.9)	26.9±4.5	0.122	23(34.3)	18(25.7)	1.2	0.351
Primary	96(70.1)	25.3±7.0		44(65.7)	52(74.3)		
**Guardian**							
Formal education	107(78.1)	26.0±6.1	0.444	52(77.6)	55(78.6)	0.0	1.000
Non-formal education	30(21.9)	24.9±7.2		15(22.4)	15(21.4)		
**Guardian income status**							
Low (<Ghc490)	100(73.0)	24.5±6.3	<0.001	47(70.1)	53(75.7)	0.5	0.564
Moderate (Ghc500-1000)	37(27.0)	29.4±4.9		20(29.9)	17(24.3)		
**Frequency of meal per day**							
Two	19(13.9)	25.3±6.4	0.692	4(6.0)	15(21.4)	6.8	0.012
Three or more	118(86.1)	25.9±6.4		63(94.0)	55(78.6)		
**Meal skipping**							
Yes	76(55.5)	26.0±6.8	0.634	42(62.7)	34(48.6)	2.8	0.122
No	61(44.5)	25.5±5.9		25(37.3)	36(51.4)		
**Food variety status**							
Low	65(47.4)	20.9±4.7	<0.001	25(37.3)	40(57.1)	5.4	0.026
High	72(52.6)	30.3±3.9		42(62.7)	30(42.9)		

Junior High School (JHS), Chi-square (X^2^), Fisher’s exact test, p<0.05, Frequency (N), percentage (%)

**Table 2:** Food variety score and food consumption score of participants.

Variables	Mean ± Sd	min—max	N (%)
Food variety score	25.8±6.4	7.0–42.0	
Food consumption score	35±5.1	18.6–49.9	
**Food consumption status**			
**Food insecure**			
Poor			1(0.7)
Borderline			66(48.2)
**Food secure**			
Acceptable			70(51.1)

Standard deviation = Sd, minimum (min), maximum (max), Frequency (N), Percentage (%).

**Table 3:** Binary logistics regression for Predictors of food consumption status.

	Food Secure					
	Unadjusted			Adjusted		
Predictors	β	OR(95%CI)	p-value	β	OR(95%CI)	p-value
**Sex**						
Male	0.858	24(1.2–4.7)	0.014	0.836	2.3(1.2–4.6)	0.017
Female		1			1	
**Community**						
Peri-urban	0.9211	2.5(1.3–5.0)	00.009	0.760	22.1(1.0–4.4)	
Rural		1			1	
**Frequency of meals per day**						
Thrice or more	1.458	44.3(1.3–13.7)	00.014	1.427	4.2(1.3–13.6)	0.018
Twice		1			1	
**Food variety status**						
High	0.806	2.2(1.1–4.4)	0.021	0.731	2.1(1.0–4.2)	0.041
Low		1			1	

β = Beta coefficient, Odds ratio (OR), p<0.05, After adjusting for age and/or sex
