# Supplementary material for: Predictors of food variety and food consumption scores of adolescents living in a rural district in Ghana
Source: PLoS One. 2023 May 31;18(5):e0286477. doi: 10.1371/journal.pone.0286477 (PMC10231777; doi:10.1371/journal.pone.0286477)
Supplement: Attachment — Submitted filename: Response to Reviewers.docx [file pone.0286477.s001.docx]

**Manuscript ID:** PONE-D-22-28451

**Response to Reviewers**

Dear Sir/Madam,

Thank you for giving us the opportunity to submit a revised draft of the manuscript titled, " PREDICTORS OF FOOD VARIETY AND FOOD CONSUMPTION SCORES OF ADOLESCENTS LIVING IN A RURAL DISTRICT IN GHANA" for publication in PLOS ONE. We appreciate the time and effort that you and the reviewers dedicated to providing feedback on our manuscript and are grateful for the insightful comments on and valuable improvements to our paper. We have incorporated suggestions made by the reviewers. Those changes are highlighted within the revised manuscript. Please see below, for a point-by-point response to the reviewers’ comments and concerns.

Reviewer(s)' comments to the Authors:

Reviewer: 1

Comments to the Author

1. Introduction – this area needs to be further developed (expand the literature review) with more articles or studies centered on adolescents’ food variety and consumption scores. You can include more information about the evidence that exists around the relevance/impact of food variety and consumption score in adolescent nutritional status, health, and growth. It would be good to see more description of the studies conducted around this with respect to adolescents specifically than in general (using adults, pregnant women, etc.) and then describe the gap with respect to food variety and consumption score in adolescents in Africa/Ghana.
   You can expand the food consumption score also which is only 4 lines on a stand-alone paragraph.

**Response: Thank you**.

Additional information has been added introduction.

The paragraph 5 in the introduction now reads:

Adolescents living in food secure household have a reduced prevalence of poor nutritional status, negative consequences of undernutrition on cognitive development and mental health disorders such as poor mood, anxiety, depression and behavioural problems [27, 28]. This psychological distress transcends into adulthood [29]. Food insecurity has also been documented to pose health challenges such as poor dietary pattern, obesity, asthma and increased the health care cost for adolescents [30-32].

2. Materials and methods –

• The authors will have to describe in detail the study area. In the topic, “Rural District” is used, which immediately causes the reader to assume the study was conducted in mainly a rural area. However, in the Abstract and Results, peri-urban residency has been used, which is not a rural area because it is a different classification on its own. The authors should be clear about what factors were taken into consideration in classifying the areas the study took place. This is connection with this statement in the Discussion – “Most of the rural settlers send their produce to urban and peri-urban areas for sale on market days, and this could have contributed to the increased food security situation among participants living in these areas.”

**Response: Thank you**

‘Rural district’ differs from ‘Rural Ghana’ or ‘rural communities.’

The rural district does not entirely mean a cluster of rural areas. What it means is that majority (55-65%) of the areas under this district is rural. Development is also very low in those areas. This particular district is very far from the regional capital, Kumasi.

This district has a capital with better or more amenities or infrastructure compared to the other areas in the district hence it is classified as peri-urban by the Asante-Akim South Municipal Assembly. The district capital cannot also be compared to the regional capital in terms of development, education, health amenities etc.

In Ghana, most of the farmers in the rural areas send their produce to the urban and peri-urban areas for sales. This will enable the farmers to buy other items that are not available in the area where they live.

Additional information has been added to the study design, area and participants (paragraph 1) under the materials and method as recommended. It now reads:

Study design, area and participants

This cross-sectional study was conducted at the Asante-Akim South municipality in the Ashanti Region of Ghana. It is one of the rural districts in Ghana with farming as the major occupation of the people. The district has land size of 0.5% of the total land in Ghana. The food crops largely cultivated are cassava, plantain, cocoyam, yam, rice and maize. About 83% of the population lives in the rural areas of the district while rest reside within the peri-urban zone of the district [33, 34].

• The authors should explain why the exclusion was made in this sentence, especially with the adolescence stage associated with poor food choices due to increased autonomy and more time spent out of the home with peers. “The food variety score was calculated from 63 selected food items. The list of foods from alcoholic and non-alcoholic beverages, sweets and snacks were excluded from the calculation of the food variety scores.”

**Response: Thank you**

Reasons for the exclusion: The food list for alcoholic and non-alcoholic beverages (such as ‘pito’, palm wine, coffee etc), sweets and snacks exempted because they were rarely or never consumed. Also, most of the participants did not provide response to the foods under this section.

Additional information has been added to the food variety score under the materials and methods. It now reads:

Food variety score

The food variety score was calculated from 63 selected food items. The list of foods from alcoholic and non-alcoholic beverages (such as ‘pito’, palm wine and coffee), sweets and snacks were excluded from the calculation of the food variety scores. The list of foods exempted were rarely or never consumed. Food item consumed at least once a week was scored ‘1’ and ‘0’ for those not consumed at all. The scores were summed to obtain the food variety score. The food variety score (FVS) was ranked ‘high’ (≥26) or ‘low’ (≤25) based on the mean food variety scores.

• “Each food group was multiplied by a weight factor.” How was the determination of this weight factor done (explain) – what is the basis of assigning the numbers or what determines the number that a food group needs to be assigned?

**Response: Thank you.**

The weight factors and its assignment to food groups were adopted from the World Food Program document (The full paper or document can be found in reference 17 in the manuscript). It is an acceptable method adopted and used by other researchers.

3. Results –
   • “A large proportion of participants were food secure; primary education (74.3%), having a guardian with formal education (78.6%), low-income status (75.7%) and not skipping meals (51.4%).” – The use of semicolon after the food secure in this sentence means it is related to the variables following it. This is incorrect for low-income status in relation to food security. Based on the literature, food security is associated with moderate to high-income status. The authors should revise the statement.

**Response: Thank you**

The semicolon has been deleted.

In terms of low-income status and food security percentage, the reviewer is encouraged to kindly make time to read from table 1.

Additional information has been added, the sentence now reads:

A large proportion of participants in the food secure category had primary education (74.3%), guardian with formal education (78.6%), low-income status (75.7%) and did not skip meals (51.4%).

• Table 4: If there is nothing interesting to talk about for the partial correlation of the food consumption score, for example the negative values or non-significance, that column should be omitted.

Response: Thank you

Additional information has been added to the results description of table 4 (paragraph 2). It reads:

The partial correlation showed a weak positive relationship between food consumption score and most of the dietary vitamins and minerals. An inverse weak correlation was found between food consumption score and pyridoxine, vitamin C and sodium. None of the relationship was significant (p<0.05) (Table 4).

4. Discussion –

• Add a reference to this sentence, “It is believed that people living in rural communities, especially farming communities, have access to a wide variety of foods compared to those in urban areas. Similarly, individuals with busy schedules may depend on fast foods which may be monotonous if they don’t take pains to plan their meals.

**Response: Thank you**

The sentence is deleted from the paragraph two of the manuscript.

• The sentence above contradicts this statement, “The association between residency and the food consumption score is confirmed by a study among pregnant women in Ethiopia [21]. Results of a recent study in Burkina Faso also supports the findings that rural or urban settlement influences food consumption [20]. One’s geographical location affects the type of food items that can be grown, be available or be accessible, and these can influence the varieties of food that may be available to them.” The authors should rephrase this statement. Again, the definition of rural, peri-urban, urban in relation to the title of the manuscript will be helpful.

**Response: Thank you**

The referred or said sentence has been deleted from the manuscript.

5. Conclusion – this section should be re-written. It is just a repetition of the same information from the Results. The authors should in addition, provide the public health implication of the study here, the strengths and weaknesses or study limitations, etc.

**Response: Thank you**

**The conclusion has been revised. It now reads:**

**Conclusion**
The study showed that majority of adolescents had acceptable food consumption scores and high food variety scores. The income status of guardian had significant impact on the food variety score of adolescents. Factors such as gender, residency, frequency of meals per day and food variety score were significantly associated with food consumption scores. The predictors of food security based on the study outcome were adolescent males, peri-urban residency, three or more frequency of meals per day and high food variety score. Governments and non-governmental organization are encouraged to implement nutrition education programmes and interventions aimed at providing adolescents with adequate nutrition knowledge, and also empower guardians economically to increase their purchasing power as it will influence their wards to have access to variety of foods for consumption and possibly increase their food consumption scores. A well-nourished adolescent is a potential healthy adult. Nutrition specific and nutrition sensitive programmes aimed at promoting adolescent health should involve guardians.

As a limitation to this study, the recall bias of the dietary intake methodology could affect the food consumption and variety scores. The subjective weight factor of the food consumption score may not be applicable for all food consumption patterns in all countries. The food consumption score is not able to show the seasonal changes in food and also measure the quantity of food gap.

**Journal Requirement**

**Response: Thank you**

Additional information has been added to the Ethics Section under the material and methods: it now reads

**Ethics**

The study protocol was approved by the Ethics Review Board of the Kwame Nkrumah University of Science and Technology, (reference number: CHRPE/AP/585/22), Ghana. Permission was granted by the Asante-Akim South Municipal Health Directorate. The purpose of the study was explained to the adolescents and guardians. Adolescents who gave their assent backed by their guardian’s consent either by signing or thumb printing on the informed consent form qualified to participate in the study.

6. Some grammatical errors sighted have been corrected.

Thank you in anticipation for a favourable response.

Yours faithfully,

Dr Michael A. Wiafe.
