# Supplementary material for: Predictors of food variety and food consumption scores of adolescents living in a rural district in Ghana
Source: PLoS One. 2023 May 31;18(5):e0286477. doi: 10.1371/journal.pone.0286477 (PMC10231777; doi:10.1371/journal.pone.0286477)
Supplement: Attachment — Submitted filename: Response to Reviewers.docx [file pone.0286477.s002.docx]

**Manuscript ID:** PONE-D-22-28451R1

**Response to Reviewers**

Dear Sir/Madam,

Thank you for giving us the opportunity to submit a revised draft of the manuscript titled, " PREDICTORS OF FOOD VARIETY AND FOOD CONSUMPTION SCORES OF ADOLESCENTS LIVING IN A RURAL DISTRICT IN GHANA" for publication in PLOS ONE. We appreciate the time and effort that you and the reviewers dedicated to providing feedback on our manuscript and are grateful for the insightful comments on and valuable improvements to our paper. We have incorporated some of the suggestions made by the reviewers and also provided some rebuttals on the comments. Those changes are highlighted within the revised manuscript. Please see below, for a point-by-point response to the reviewers’ comments and concerns.

Reviewer(s)' comments to the Authors:

Reviewer: 1

Comments to the Author

1. Mention the country name in the abstract (method) section of the manuscript.

**Response: Thank you**.

Additional information has been added introduction.

The country’s name has been mentioned in the abstract as suggested. It is highlighted in	the manuscript

2. Please don't use the same words as the study's keywords, which are already in the title of the	paper.

**Response: Thank you**

keywords are words provided to make the article easily discoverable and must also be related to the work. Among the keywords already provided, the authors want to know which of them are outside the domain of the article or the work?

The reviewer(s) can suggest alternative keywords and if they worth being used as keywords by the authors we will fix it/them in the manuscript.

3. Please describe the gap in the prior literature and the contribution that this study intends to	make. Additionally, please include the contribution of this study in the literature.

**Response: Thank you**

The last paragraph of the introduction addresses the comments 3. There is paucity of knowledge about food variety and food consumption scores of adolescents in rural communities in Ghana. Additional information has also been added to the last paragraph of the introduction.

4. I could not find the hypotheses of the study.

**Response: Thank you.**

5. I could not find the details of the study beneficiaries in the manuscript.

**Response: Thank you.**

Information about the study beneficiaries are provided under the study design, area and participants of the materials and method section.

6. Why did you select the Asante-Akim South municipality in the Ashanti Region of Ghana? Please talk about how important it is for Ghana's food security. Also discuss the other characteristics of the study area.

**Response: Thank you**

The reasons for the selection of the Municipality is based on the title (living in rural district) and dwellings of the percentage (83%) of the population.

The information under the study design, area and participants of the materials and methods section clearly explains the comment 6.

The reviewer(s) should kindly indicate the specific characteristics of the study area that need to be added .

7. How did you estimate the total sample size (137)? Did you use any sampling formula to reach this sample size?

**Response: Thank you**

The sample size used for this study was that calculated for an intervention study. The data used for this paper was collected as part of baseline data of an intervention study. For the calculation of the sample size, assess the published protocol via https://doi.org/10.1177/02601060221074433

8. Explain the sampling section criteria in more detail.

**Response: Thank you**

Additional information has been added to the sampling technique in the manuscript. It now reads:

A total of one hundred and thirty-seven (137) healthy adolescents aged 10-14 years were selected using a multi-stage sampling method from the municipality for this study.

9. I am wondering about the time period during which food related information was obtained.

**Response: Thank you**

Since this study is cross-sectional all information were collected once and at the same time.

10. Please explain your questionnaire in your revised manuscript.

**Response: Thank you**

The authors would want to know how the reviewer(s) want the questionnaire to be explained?

For instance:

1) how many times do you eat a day? 1. once	2. twice
2) Age: …………..
3) Sex: 1. male	2. female
4) Do you skip any meal? 1. Yes	2. No

11. How did you check the reliability of the data collection instrument?

**Response: Thank you**

A pilot study was conducted with the questionnaire before the actual data collection.

12. Explain in full detail how food scores were calculated.

**Response: Thank you**

Additional information has been added to the calculations of the food scores in the manuscript.

13. Please explain how you decided to use binary logistic regression. Put its formulas together and explain the explanatory and dependent variables.

**Response: Thank you**

Binary logistic regression is a regression model where the target variable is binary, that is, it can take only two values, (0 or 1) or (Yes or No). The analysis was not done manually hence provision of the formula may not be necessary. SPSS software was used in the data analysis.

The independent variables or predictors were sex, community, frequency of meals per day and food variety status. The dependent or outcome variable was food secure.

14. The ethics section could be taken out of the manuscript and placed with other statements, according to the journal's rules.

**Response: Thank you for bringing it to our attention**

15. A subheading "descriptive statistics" should be added immediately after "Results."

**Response: Thank you**

Recommendation has been implemented in the manuscript.

Other sub-headings have also been introduced in the results to maintain consistency.

16. The interpretation of descriptive statistics needs extensive revision. Please revise it. Look for high-quality papers to help you with your writing.

**Response: Thank you**

Some revisions have been made as requested in the descriptive statistics. The authors request the reviewer(s) to be kind to indicate any other specific revision.

17. The legend in Table 1 should be revised as it does not present an association.

**Response: Thank you**

The authors do not agree entirely. The chi-square test is a test of association.

A revision has been made in the heading for Table 1.

18. Please present the results of binary logistic regression in a proper way. Get help from relaxant literature. What is the reason for selecting only four variables in the logistic model? The interpretation of the model results is not enough.

**Response: Thank you**

The position of the constant (1) has been changed in Table 3.

The authors wanted to know whether the same factors that produced significant relationship between food consumption score were also able to significantly predict food security. Hence the four variables.

19. How did you estimate micronutrients? No information is provided about it in the methodology section, or I am missing something (Table 4).

**Response: Thank you bring this to our attention**

Additional information has been added to the dietary intake under the materials and methods sections. It reads:

A 24-hour recall was used to collect three days (one weekend and two working days) dietary data. Participants were asked to tell us how many times (breakfast, mid-morning snack, lunch, mid-afternoon snack, dinner and bed-time snack) they eat a day and the detailed of the food consumed. Participants were shown food handy measures to estimate how much of the food consumed at each meal. Each food was converted to grams and the nutrients were estimated using the Nutrient Analysis Template developed by the University of Ghana. The averages for the nutrients were recorded.

20. I could see a number of limitations with the study. Why did the author not include it in the manuscript?

**Response: Thank you**

Currently, the few limitations identified were the ones written in the manuscript. The authors are open to further suggestions on limitations by the reviewer(s).

21. Most of the discussion is repeating the results. Please discuss the results by repeating them.

**Response: Thank you**

In discussion, it is expected that the main outcome(s) of the current study is stated and compared or contrast with other studies findings. Probably given reasons for the otherwise of contrary outcomes between the current and the previous or other(s) studies.

The authors did exactly the suggestion above in the manuscript, however the authors are ready to accept suggestion(s) from the reviewer(s) that will add value to the discussion.

Additional information has been added to the discussion and highlighted.

22. The table data presentation is totally absurd. Improve the table data presentation.

**Response: Thank you**

The reviewer(s) must be specific about the specific table(s) that is/are not well presented, and the authors will be ready to address it.

Some grammatical errors sighted have been corrected.

Thank you in anticipation for a favourable response.

Yours faithfully,

Michael A. Wiafe, PhD.
